# 828. Dermal Substitutes as a Carrier for Mechanical SVF

**DOI:** 10.1093/jbcr/irag033.267

**Published:** 2026-04-06

**Authors:** Bong-Sung Kim

**Affiliations:** Zurich University Hospital, Zurich, Zurich

## Abstract

**Introduction:**

Contemporary management of full-thickness burn defects frequently incorporates dermal substitutes, with clinicians selecting from a wide array of commercially available options that differ in composition, structure, and biological properties. Cell-based therapies, particularly those utilizing adipose tissue-derived progenitor cells, have emerged as promising adjuncts recently. Mechanical isolation methods, such as mechanical stromal vascular fraction (mSVF), offer a practical, regulatory-compliant approach for obtaining regenerative cells. This study evaluated the feasibility of seeding mSVF onto various commercially available dermal substitutes in vitro to their biocompatibility.

**Methods:**

Mechanical SVF was isolated from human donors obtained during elective surgeries using a standardized protocol involving emulsification and centrifugation steps. The mSVF was then seeded onto multiple commercial dermal substitutes under standard culture conditions. Supernatants were analyzed for total protein content and growth factor concentrations. Cellular integration was assessed via histological examination and electron microscopy.

**Results:**

Significant variability was observed in mSVF ingrowth among the tested dermal substitutes. Rigid substitutes with dense surfaces exhibited poor cellular infiltration, while others with a greater pore size fostered integration within 1–2 weeks. Biological dermal substitutes demonstrated higher protein and growth factor levels in supernatants.

**Conclusions:**

The interaction between mSVF and commercial dermal substitutes varies substantially, influencing cellular engraftment and secretory profiles. These findings highlight the importance of scaffold selection in tissue engineering strategies with mSVF and suggest potential clinical implications for optimizing regenerative outcomes in burn reconstruction.

**Applicability of Research to Practice:**

The combination of FDA-approved mSVF protocols and commercially available dermal substitutes may be an interesting avenue to treat critical wounds in the future.

**Funding for the study:**

The research was funded by the Novartis Foundation for Medical-Biological Research.

